# Motivating online language learning: exploring ideal L2 self, grit, and self-efficacy in relation to student satisfaction

**DOI:** 10.3389/fpsyg.2023.1293242

**Published:** 2023-11-13

**Authors:** Zhijie Sun, Bingyu Mu

**Affiliations:** ^1^Department of Foreign Language, Lanzhou University of Finance and Economics, Lanzhou, China; ^2^Zhengzhou Institute of Technology, Zhengzhou, China

**Keywords:** online learning, EFL students, ideal L2 self, L2 grit, online learning self-efficacy, online learning satisfaction

## Abstract

**Introduction:**

This study delves into the intricate network of motivational factors that influence online learning satisfaction among intermediate-level English as a Foreign Language (EFL) students in mainland China.

**Methods:**

A diverse sample of 496 EFL students participated in this research. Structural Equation Modeling was employed as the analytical method.

**Results:**

The results of the study reveal significant and positive relationships between ideal L2 self and L2 grit with online learning satisfaction. Additionally, online learning self-efficacy emerged as a crucial mediator between ideal L2 self and online learning satisfaction, as well as between L2 grit and online learning satisfaction.

**Discussion:**

These findings provide valuable insights into the motivational dynamics within online language learning contexts. They offer practical implications for educators and instructional designers seeking to enhance students’ online learning experiences.

## 1. Introduction

In recent years, the ubiquitous presence of digital media has reshaped how individuals live their lives, communicate, and engage with information, particularly among younger generations (Simsek et al., 2021; Su and Guo, 2021; Lei et al., 2022; Pangarso and Setyorini, 2023). This cohort, often referred to as “digital natives,” represents the first generation to grow up with seamless access to the Internet and handheld digital devices. While the transformative effects of digital media on leisure activities, entertainment preferences, and social interactions have been extensively studied (Jastrow et al., 2022; Pikhart et al., 2022; Liu et al., 2023), the impact of digital media on second language (L2) learning remains an evolving area of exploration, warranting further research and investigation.

As far as digital education is concerned, numerous factors influence students’ satisfaction with the online learning experience. Notably, the quality of instruction plays a pivotal role in shaping students’ contentment (Kuo et al., 2014; Landrum et al., 2021; Pikhart et al., 2022). As Gopal et al. (2021) emphasize, educators delivering courses in online formats must possess a profound understanding of the psychological dynamics at play among their students to ensure effective teaching. This involves aligning educational objectives with students’ needs, developing clear and accessible course materials, and providing timely feedback, among other considerations.

Concurrently, the socio-educational paradigm, pioneered by Gardner (1985) and Masgoret and Gardner (2003), has long been a dominant hypothesis in L2 motivation studies. Central to this paradigm is the concept of “integrativeness,” which reflects the inclination of L2 learners to become part of the community and culture associated with the target language. However, contemporary scholars have begun to question the continued relevance of core ideas within the socio-educational paradigm, including integrativeness and motivation, within the context of modern L2 learning environments (Dörnyei, 2009; Csizér, 2019). In response to these shifts in perspectives and paradigms, there has emerged a growing demand for a more suitable conceptualization of L2 motivation that aligns with the realities of a globalized world (Ushioda, 2006; Ushioda and Dörnyei, 2009). Addressing this need for a contemporary framework, Dörnyei (2005) and others introduced the L2 Motivational Self System (L2MSS). This paradigm posits that language learners are most motivated when they can vividly envision their “ideal L2 self” in the future and are driven to bridge the gap between their current self and this idealized L2 self-image (Ueki and Takeuchi, 2013). In essence, a robust L2 learning motivation is intricately tied to the desire to diminish the disparity between one’s present language abilities and the vision of their ideal L2 self (Fathi et al., 2023).

Additionally, this study explores the concept of “grit” as a third crucial variable. Grit has been introduced in the literature as a potent predictor of academic success (Datu et al., 2017; Teimouri et al., 2022) and has demonstrated associations with positive emotions such as life satisfaction and psychological wellbeing (Vainio and Daukantaitë, 2016; Jiang et al., 2020). These prior investigations have suggested that gritty individuals are more inclined to attribute positive outcomes to their efforts and maintain a growth-oriented perspective, both of which contribute to heightened positive emotions (Von Culin et al., 2014; Hill et al., 2016; Credé et al., 2017; Fong and Kim, 2021). Consequently, examining the relationship between L2 grit and online learning satisfaction becomes particularly pertinent, given the potential influence of grit on emotional wellbeing in the context of language learning (Elahi Shirvan et al., 2021; Zarrinabadi et al., 2022).

Finally, the study delves into the domain of “online learning self-efficacy” as the fourth variable under investigation. Self-efficacy, a term introduced by Bandura (1977), refers to an individual’s assessment of their own capabilities to successfully complete a task. In the context of online education, where students navigate a novel and technologically mediated learning environment, self-efficacy becomes a pivotal factor. As highlighted by Zimmerman and Kulikowich (2016), online learning self-efficacy (OLSE) serves as a gauge of a student’s potential for success in online learning settings. While previous OLSE research has predominantly focused on its technological dimensions (Hodges, 2008), this study probes deeper, exploring the intricate interplay between students’ ideal L2 selves, L2 grit, online learning self-efficacy, and their ultimate satisfaction with the online learning experience.

In light of these considerations, the overarching objective of this research is to investigate the relationships between ideal L2 self, L2 grit, and online learning self-efficacy as predictors of online learning satisfaction among English as a Foreign Language (EFL) students in mainland China. Although previous research has examined various factors affecting language learning motivation and satisfaction, a notable gap exists in understanding the interplay between these specific constructs within the context of online language learning, particularly among EFL students in mainland China. This study aims to bridge this gap by delving into the intricate motivational dynamics that influence online learning satisfaction. We seek to unravel how EFL students’ aspirations related to their ideal L2 selves, their levels of tenacity (L2 grit), and their confidence in navigating the online learning environment (online learning self-efficacy) collectively shape their satisfaction with online language courses. This comprehensive examination addresses the unique challenges and opportunities presented by online language education, where the shift from traditional classrooms to virtual platforms introduces novel motivational factors.

The significance of this study lies in its potential to inform educational practices and instructional design strategies tailored to the specific needs of EFL students engaged in online language learning. By shedding light on the pivotal role played by ideal L2 self, L2 grit, and online learning self-efficacy in fostering satisfaction, educators and course designers can gain insights into how to create more motivating and effective online language courses. These insights can contribute to the improvement of online learning experiences, potentially increasing students’ motivation, persistence, and ultimately, their language proficiency. Additionally, as online learning continues to play a significant role in contemporary education, understanding the factors that enhance satisfaction is of paramount importance for educational institutions striving to provide high-quality language education in virtual settings.

## 2. Literature review

### 2.1. L2 motivational self-system

Dörnyei’s L2 Motivational Self System (2009) stands as a significant theoretical framework that combines contemporary ideas about L2 motivation with insights from behavioral psychology concerning potential selves. This comprehensive model introduces the notion of “future self-guides,” encompassing both ideal and ought selves, which wield substantial influence over language learning behaviors.

The ideal self, according to Higgins (1987), represents the attributes an individual aspires to possess, reflecting their hopes, dreams, and desires. On the other hand, the ought self-embodies the attributes one believes they should or must possess, encapsulating a sense of duty, obligation, or responsibility (Higgins, 1987, p. 320). Within the L2MSS, three principal components intricately shape L2 motivation. Firstly, the Ideal L2 Self, as defined by Dörnyei (2009), stands as the core element representing the L2-specific facet of an individual’s ideal self. It embodies the vision of a proficient language user, reflecting the learner’s aspirations and desires for language proficiency. Secondly, the ought-to L2 Self pertains to the attributes one believes they ought to possess to meet expectations and avoid potential negative outcomes. It reflects a sense of responsibility and duty associated with achieving language proficiency. Thirdly, the L2 Learning Experience component encompasses motives situated in the immediate learning environment. It incorporates executive functions related to the learning experience, encapsulating the learner’s interaction with the learning context, materials, and activities, which significantly influence their motivational stance (Dörnyei, 2009, p. 29).

Dörnyei highlights that future self-guides, while related to future end-states, extend beyond mere goals by encompassing cognitive, emotional, visual, and sensory dimensions. These self-guides provide a vivid, multifaceted representation of the learner’s envisioned future self, exerting a profound impact on motivation. To maximize the motivational potential of future self-guides, Dörnyei and Ushioda (2011) outline nine critical requirements. Firstly, an idealistic future self-image is crucial; the learner must possess a vivid and ambitious image of their future L2 self (Nourzadeh et al., 2023). Secondly, separation from the present self is essential, emphasizing growth and development. Thirdly, the accessibility of the future self-image is paramount; it must be easily accessible and readily available for mental projection. Fourthly, learners must find their future self-guides acceptable and congruent with their personal aspirations. Fifthly, the future self-image should not be perceived as overly certain, allowing room for growth and adaptability. Sixthly, a harmonious interplay between the ideal and ought selves ensures a balanced motivational orientation. Seventhly, future self-guides must be activated and brought to the forefront of the learner’s consciousness. Eighthly, concrete steps and strategies should be in place to facilitate the realization of the future self. Lastly, recognizing and addressing potential fears or barriers is essential to maintaining a positive motivational stance (Dörnyei and Ushioda, 2011).

This framework provides a nuanced understanding of the intricate interplay between learners’ future self-guides and their motivation in the context of language learning. It integrates cognitive, emotional, and perceptual dimensions, offering valuable insights into the motivational processes that underlie language acquisition. From this perspective, the L2 Motivational Self System provides a powerful lens through which to explore the dynamic relationship between learners’ idealized selves, their obligations, and the contextual influences that shape their language learning process.

### 2.2. Online learning satisfaction

Online learning satisfaction stands as a pivotal determinant of educational success, encapsulating the alignment between students’ expectations, actual experiences, and a sense of fulfillment (Bervell et al., 2020; Pikhart et al., 2022). In the realm of virtual education, this diverse mindset emerges as a consequential outcome when incorporating novel technologies in instructional settings (Jung, 2014; Zou et al., 2022). Landrum et al. (2021) emphasize that online learning satisfaction encompasses a nuanced comprehension of how curriculum objectives align with students’ daily lives and the broader academic environment, alongside their perceptions of the instructor. This perception of satisfaction plays a vital role in both the adoption and quality of online courses, as noted by Sampson et al. (2010). Notably, the Online Learning Consortium recognizes learning satisfaction, alongside measures of learning effectiveness, instructor satisfaction, scales of assessment, and accessibility, as integral components for evaluating the educational value of online courses.

Previous research has delved into an array of variables that pertain to technology, educational methodologies, enjoyment, and self-motivation, all of which influence student satisfaction with online courses (Brinkerhoff and Koroghlanian, 2007; Eichelberger and Ngo, 2018; Bolliger and Martin, 2021; Derakhshesh et al., 2022). For instance, Jiang et al. (2021) scrutinized the influence of perceived utility and ease of use on higher education students, revealing both to be pivotal predictors of online course satisfaction, with perceived utility demonstrating greater predictive power. Similarly, studies have identified that the pleasure derived from online experiences hinges on both perceived utility and ease of use. In a structural equation modeling study, She et al. (2021) explored the intricate interplay between student satisfaction, online learning, involvement, and self-efficacy. Bervell et al. (2020) uncovered that three types of interactions—learner-learner, learner-teacher, and learner-material—significantly impacted students’ satisfaction with online courses.

Course layout, according to Eom et al. (2006), wields a substantial influence on student satisfaction in online college education. Moreover, Cobb (2011) emphasized that social presence profoundly affects nursing students’ overall satisfaction with their educational process. This sentiment is corroborated by Strong et al. (2012), who established that social interaction and the virtual learning environment significantly contribute to learners’ satisfaction. Marks et al. (2005) further assert that perceived educational outcomes wield a considerable influence on student satisfaction in online courses. Cohen and Baruth (2017) proposed that openness to new experiences and conscientiousness serve as antecedents to course contentment, aiming to probe the link between students’ character traits and their engagement in online courses. In an extensive cross-country study, Baber (2020) concurrently examined antecedents to students’ satisfaction with course content and their perceptions of educational quality, identifying course structure, motivation, in-class communication, instructor expertise, and support as pivotal factors.

Wu et al. (2010) contributed a hypothesized model of learning satisfaction encompassing six variables. The research findings underscored that performance expectations and learning environments emerged as the primary indicators of learning satisfaction, with all other characteristics exerting an indirect influence. Understanding and assessing online course satisfaction is of paramount importance, as it serves as a critical barometer for student and program outcomes (Willging and Johnson, 2009). Prior research underscores that factors such as instructional quality, dropout rates, commitment to course completion, learning achievements, and the consistency of online learning all play pivotal roles in influencing student satisfaction (Eom et al., 2006; Park and Choi, 2009; Alebaikan and Troudi, 2010; Ke and Kwak, 2013; Lee et al., 2013; Kuo et al., 2014; Parahoo et al., 2016; Lin et al., 2017; Aldosemani et al., 2019).

Overall, the literature on online learning satisfaction provides valuable insights into the multifaceted nature of this crucial aspect of educational success. It encompasses the alignment between students’ expectations, experiences, and a sense of fulfillment, all of which are essential for the adoption and quality of online courses. Understanding online learning satisfaction is particularly important in the context of integrating novel technologies in instructional settings, as it directly influences both student and program outcomes. Previous studies have explored various factors influencing satisfaction, including perceived utility and ease of use, interactions with peers, teachers, and course materials, course layout, social presence, and perceived educational outcomes. This extensive body of research underscores the significance of assessing and understanding online course satisfaction, as it serves as a critical barometer for educational success in virtual learning environments.

### 2.3. Grit

Since its invention by Duckworth et al. (2007), grit—perseverance and passion for long-term and higher-order goals—has drawn increasing attention from academics and the public. As claimed by Duckworth et al. (2007), “grit entails working strenuously toward challenges, maintaining effort and interest over years despite failure, adversity, and plateaus in progress” (pp. 1087–1088). According to Shechtman et al. (2013), the US Department of Education has lately stressed the importance of grit, tenacity, and perseverance in determining a student’s performance in the twenty-first century. To encourage learners’ grit in the classroom, a number of educational institutions and groups have started creating and utilizing instructional materials and programs (Shechtman et al., 2013; Dweck et al., 2014; Laursen, 2015). Grit adds additional predictive value for performance criteria over and above natural or inherent competence, and it is just as essential as skill in determining a student’s success (Duckworth et al., 2007, 2021; Li et al., 2018).

Consistency of interest and perseverance of effort are two lower-order components that make up grit, as defined by Duckworth et al. (2007). Consistency of interest relates to an individual’s consistency of enthusiasm for a more complex objective, even when facing challenges, hurdles, or setbacks, as opposed to perseverance of effort, which relates to an individual’s propensity to spend enduring energy over a lengthy amount of time. Age can additionally boost grit. As people age, they may come to value hard work and persistence in pursuit of a particular goal more (Duckworth et al., 2007; Duckworth and Quinn, 2009). Additionally, it has been discovered that grit is not connected to an individual’s gender, race, or academic standing (Credé et al., 2017; Credé, 2018; Ponnock et al., 2020), as well as to general intellect and physical health (Hill et al., 2016; Fong and Kim, 2021).

However, it is noteworthy to mention that there has been ongoing debate regarding the measurement of grit. While the two-factor structure proposed by Duckworth et al. (2021) has gained attention, studies such as those conducted by Sulla et al. (2018) and Postigo et al. (2021) have presented evidence for a one-dimensional structure. This controversy has been acknowledged by the authors themselves in recent discussions. These varying perspectives highlight the complexity and ongoing discourse surrounding the certain aspects of grit. Grit is distinct from the desire for success. The urge for achievement, as defined by McClelland (1961), shows an individual’s propensity for finishing tasks that result in rapid feedback on their performance, particularly those that are neither too easy nor too tough. On the other hand, tenacious people purposefully set themselves ambitious objectives and stick to them in the face of challenges and setbacks (Duckworth et al., 2007; Von Culin et al., 2014). Grit and resilience should be separated. While grit and resilience both encompass a tendency to withstand failure, gritty people are committed to a long-term goal for a lengthy period of time: “Grit is not just having resilience in the face of failure, but also having deep commitments that you remain loyal to over many years” (Perkins-Gough, 2013, p. 16). Self-control, a Big Five conscientiousness subcomponent that has been found to be strongly correlated with grit (Credé et al., 2017), has also been claimed to be separate from grit. Although grit and self-control both include protecting one’s objectives against challenges and impulses, Duckworth et al. (2007) claimed that the difference between the two lies in the nature of the goals being guarded. Self-control involves defending one’s relatively short-term goals, while grit is about concentrating on one’s long-term objectives.

Despite disappointments and losses, tenacious people persistently pursue their ultimate aim (Duckworth and Gross, 2014; Werner and Milyavskaya, 2019). However, those with self-control must arbitrate between lower-level objectives, which may occasionally necessitate acting in a contradictory manner. Although the two traits are highly associated, grit should be separated from the Big Five traits of conscientiousness (Credé et al., 2017; Ponnock et al., 2020). Grit focuses one’s industrious character toward a long-term objective, whereas conscientiousness stresses one’s hardworking nature. In other words, what sets grizzled people apart from just hardworking people is their tenacity (Duckworth et al., 2007; Abuhassàn and Bates, 2015). Another trait of grit that is lacking in conscientiousness is the ability to maintain one’s passion for a long-term objective despite obstacles and setbacks (Elahi Shirvan et al., 2021; Teimouri et al., 2022).

In summary, grit extends beyond inherent competence, providing additional predictive value for various performance criteria and proving to be as crucial as skill in shaping students’ success. This multifaceted trait comprises lower-order components, including consistency of interest and perseverance of effort, which further contribute to its significance. In essence, grit’s essence lies in its tenacity, setting individuals with this trait apart from those characterized solely by industriousness. These distinctions emphasize the unique and vital role that grit plays in shaping individuals’ educational journeys and personal growth. However, while there is a wealth of research on the importance of grit in various contexts, a significant gap remains in understanding its specific role in the context of online language learning satisfaction among EFL students. This study seeks to address this gap by exploring how grit influences online learning satisfaction and whether it does so directly or through the mediation of online learning self-efficacy, thus contributing to a more comprehensive understanding of the motivational factors affecting EFL students’ online learning experiences.

### 2.4. Online learning self-efficacy

Self-efficacy, a central construct in social cognitive theory (Bandura, 1977), has been identified as a critical determinant of students’ academic success across various educational contexts. Its significance is particularly pronounced in the domain of language learning and teaching, where it serves as a potent predictor of language learning strategies, motivation, and achievement (Bandura, 2001; Quang et al., 2022). Several studies have underscored the positive correlation between high academic self-efficacy and academic performance. For instance, research by Chen and Lin (2009) revealed that students with elevated academic self-efficacy tend to outperform their peers with moderate to low self-efficacy beliefs. This connection between self-efficacy and academic achievement extends to challenging and innovative learning environments, such as online education (Wong and Bakar, 2009; Chou et al., 2023).

Low self-efficacy can have detrimental effects on students in online learning settings and may even lead to dropout in certain instances (Lee et al., 2013). Therefore, understanding and nurturing online learning self-efficacy (OLSE) is of paramount importance for both educators and learners engaged in online courses. OLSE is inherently context-sensitive, shaped by the interplay of three key factors: technology, learning processes, and social interaction (Shen et al., 2013). Shen et al. (2013) identified six core characteristics of OLSE that significantly influence students’ ability to successfully complete online courses. These characteristics encompass peer interactions, instructor interactions, self-regulation, course administration systems, peer socialization, and time management, online learning, and technology use, as proposed by Zimmerman and Kulikowich (2016).

In practical terms, OLSE can be defined as learners’ perception of their capacity to learn and accomplish tasks in online learning environments. This perception is influenced by a multitude of factors, including psychological, emotional, cognitive, environmental, and social elements (Cho and Cho, 2017; Kara et al., 2021). Moreover, the perceived OLSE of language learners can play a pivotal role in mediating the relationship between grit and engagement, as highlighted by Shen et al. (2013). This suggests that enhancing OLSE can contribute to more resilient and engaged language learners in online settings.

Furthermore, research conducted by Womble (2007) demonstrated a positive correlation between online self-efficacy and course satisfaction. Learners who exhibit higher levels of online self-efficacy tend to report greater satisfaction with their online learning experiences. This underscores the far-reaching impact of OLSE on learners’ overall course perceptions and highlights its significance in online education. It is important to note that a student’s online self-efficacy can be influenced by various individual factors, including gender, academic standing, and prior online learning experiences (Artino, 2009; Zhu et al., 2020; Stephen and Rockinson-Szapkiw, 2021). Acknowledging these individual differences is crucial when designing effective online language learning experiences and support mechanisms.

Taken together, the perceived OLSE of language learners can serve as a significant mediator in the relationship between grit and engagement, suggesting that enhancing OLSE can foster more resilient and engaged language learners in online settings (Alqurashi, 2016). Importantly, empirical evidence demonstrates that higher levels of OLSE are associated with greater satisfaction with online learning experiences, highlighting its pervasive influence on students’ overall course perceptions (Derakhshesh et al., 2022). It is essential to recognize that individual factors, such as gender, academic standing, and prior online learning experiences, can shape a student’s OLSE. Therefore, accounting for these individual differences is imperative when designing effective online language learning experiences and support mechanisms.

### 2.5. The study hypotheses

**FIGURE 1:**
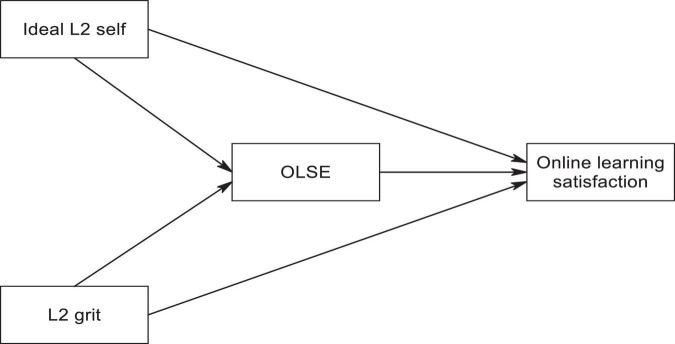
The hypothesized model.

In this section, we outline our hypothesized model (see Figure 1) and present four key hypotheses (H1, H2, H3, and H4) that underpin our investigation into the relationships between ideal L2 self, L2 grit, online learning self-efficacy, and online learning satisfaction. These hypotheses draw from well-established theoretical frameworks and existing empirical evidence to provide a foundation for our research.

Hypothesis 1 (H1): *Ideal L2 self will positively predict online learning self-efficacy.*

This hypothesis aligns with Bandura’s Social Cognitive Theory (1989), which emphasizes the significance of self-beliefs, such as self-efficacy, in influencing motivation and performance. The ideal L2 self represents learners’ aspirations and desires concerning their future language proficiency (Adolphs et al., 2018; Zhang et al., 2022). Drawing from Bandura’s theory, it is reasonable to propose that individuals who envision themselves as proficient language users (ideal L2 self) are more likely to believe in their capability to excel in online language courses (online learning self-efficacy). This alignment of self-beliefs resonates with Bandura’s theory, which underscores the interplay between self-concept and motivation. Furthermore, the study by She et al. (2021) found a significant relationship between self-efficacy and satisfaction in the context of online learning, providing empirical support for this hypothesis.

Hypothesis 2 (H2): *L2 grit will positively predict online learning self-efficacy.*

Grit, characterized by perseverance and passion for long-term goals, implies a sustained effort to overcome challenges and obstacles. Such a disposition closely relates to self-efficacy, as individuals with high levels of grit are more likely to believe in their ability to overcome difficulties in the pursuit of their long-term objectives, akin to the concept of self-efficacy (Usher et al., 2019; Teimouri et al., 2022). Recent investigations have further emphasized the significant interplay between grit and self-efficacy in online learning contexts. For instance, Sulla et al. (2022) found that grit played a pivotal role in university students’ online learning experiences during the COVID-19 pandemic, highlighting its impact on academic performance. Similarly, Yang et al. (2022) conducted a cross-cultural study, demonstrating the substantial contribution of academic buoyancy and self-efficacy to L2 grit among English language learners.

Also, Duckworth’s seminal work on grit (Duckworth et al., 2007) provides a theoretical basis for this hypothesis, as individuals high in grit are expected to possess a strong sense of self-efficacy in online learning due to their determination and persistence (Malureanu et al., 2021). Some existing studies in the literature (e.g., Baber, 2020; Paradowski and Jeliñska, 2023; Zhao et al., 2023) highlight the importance of students’ character qualities, such as perseverance, in their satisfaction with online courses, further reinforcing this hypothesis.

Hypothesis 3 (H3): *Online learning self-efficacy will mediate the relationship between ideal L2 self and online learning satisfaction.*

This hypothesis is consistent with Bandura’s Social Cognitive Theory (1977), which posits that self-efficacy beliefs play a mediating role between personal goals and outcomes. Within the context of our study, we propose that ideal L2 self, representing learners’ goals and aspirations in language proficiency, will exert its influence on online learning satisfaction through online learning self-efficacy. Students who have confidence in their ability to succeed in online language courses are more likely to exhibit effective learning behaviors and, as a result, experience higher satisfaction with their online learning process (Bao et al., 2021; Derakhshesh et al., 2022). The findings of Womble (2007) and She et al. (2021) support the idea that self-efficacy mediates the relationship between learners’ characteristics and online learning satisfaction.

Hypothesis 4 (H4): *Online learning self-efficacy will mediate the relationship between L2 grit and online learning satisfaction.*

This hypothesis builds upon the previous one by focusing on the mediating role of online learning self-efficacy in the relationship between L2 grit and online learning satisfaction. Given that grit encompasses perseverance and passion for long-term goals, it is reasonable to assume that individuals high in grit will exhibit higher levels of online learning self-efficacy (Derakhshan and Fathi, 2023). This, in turn, should positively impact their online learning satisfaction (Shen et al., 2013). Through this mediating relationship, online learning self-efficacy serves as the conduit through which the enduring determination and effort associated with grit translate into higher satisfaction with the online learning experience (Hodges, 2008; Alqurashi, 2016). The studies by Cohen and Baruth (2017) and Derakhshan and Fathi (2023) propose that character traits, including perseverance, are antecedents to course satisfaction, further supporting the idea that grit influences online learning satisfaction indirectly through self-efficacy.

## 3. Materials and methods

### 3.1. Participants

The study included 496 intermediate-level EFL students from diverse regions across mainland China, spanning various demographic and academic backgrounds. Participants’ ages ranged from 19 to 27 years (*M* = 20.88, *SD* = 2.07), aligning with the typical age range observed among university students in China. The sample had a fairly balanced gender distribution, with 189 male students and 307 female students, reflecting the evolving gender diversity in Chinese higher education. These students typically pursue specialized language courses in addition to their major field of study at the university level. This online intermediate-level EFL course is designed to enhance their proficiency in English, a skill of increasing importance in the global academic and professional landscape. The participation in such courses is typically a mandatory component of their academic curriculum. Furthermore, our participants hailed from various provinces and cities across mainland China, encompassing both urban metropolises and rural locales. This deliberate inclusion aimed to account for potential regional variations in online learning experiences, as approximately 58% were from urban areas and 42% from rural regions.

In terms of academic background, our participants represented a wide array of fields of study, including sciences (27%), humanities (24%), engineering (18%), and social sciences (31%). This diversity in academic pursuits provides a robust foundation for comprehensively exploring the study’s variables within various educational contexts. The sample also reflected the ethnic and cultural diversity within China, including students from various ethnic backgrounds, such as Han Chinese (78%) and various minority ethnic groups (23%), mirroring the multicultural landscape of contemporary Chinese universities. All participants were actively pursuing intermediate-level EFL studies, indicating a fundamental level of English language proficiency. They had varying experiences with online learning, participating in a wide range of online courses across different subjects. This diversity in online learning experiences enriched the study by examining how individual characteristics intersected with online learning satisfaction across diverse course contexts.

Also, ethical considerations were rigorously followed throughout the research process, including obtaining ethical approval from the Department of Foreign Language, Lanzhou University of Finance and Economics. We also ensured that informed consent was secured from all participants in accordance with established ethical guidelines.

### 3.2. Instruments

#### 3.2.1. Ideal L2 self

To assess the ideal L2 self of EFL learners, we employed an 8-item scale originally developed by Papi and Abdollahzadeh (2012). An illustrative item from this construct reads as follows: “I can envision myself residing abroad and effectively utilizing English to communicate with locals.” Respondents provided their evaluations using a six-point Likert scale, which ranged from 1 (strongly disagree) to 6 (strongly agree).

#### 3.2.2. Grit

We gauged the grit of EFL learners using the L2 Grit Scale, which was validated by Teimouri et al. (2022). This scale comprises 9 items distributed across two subscales: Perseverance of Effort (POE) with 5 items (e.g., “I invest substantial time and effort into enhancing my weaknesses in English language”) and Consistency of Interest (COI) with four items (e.g., “I was fervently dedicated to learning English in the past but have gradually lost interest”). Respondents rated these items on a five-point Likert scale, ranging from 1 (strongly disagree) to 5 (strongly agree).

#### 3.2.3. Online learning self-efficacy (OLSE)

To evaluate learners’ online learning self-efficacy, we adapted a questionnaire from the Online Learning Self-Efficacy Scale, initially compiled by Xie et al. (2011). This questionnaire comprised 14 items designed to assess participants’ perceptions of their competence, environmental control, and behavioral control when undertaking online learning tasks. An example item from this questionnaire was: “I can effectively address most of the challenges that arise during online learning.” Each item was rated on a five-point Likert scale, ranging from 1 (completely disagree) to 5 (completely agree). Higher scores indicated a higher level of online learning self-efficacy.

#### 3.2.4. Online learning satisfaction

The measurement of students’ online learning satisfaction was adapted from a four-item scale originally developed by Lin (2005). In the context of this study, the term “course” was substituted with “online learning” in the original scale. For instance, one item was rephrased as follows: “The online learning activities aligned with my expectations regarding what I aspired to learn.” Participants were required to provide their responses on a five-point Likert scale, ranging from 1 (strongly disagree) to 5 (strongly agree).

### 3.3. Procedure

The participant recruitment process was meticulously executed with a deliberate focus on diversity and representation. Initially, potential participants were identified across various university campuses within mainland China. To ensure broad representation encompassing academic disciplines, geographic regions, and demographic backgrounds among intermediate-level EFL students, a purposive sampling technique was thoughtfully employed.

To establish contact with prospective participants, a collaborative effort was initiated with university administrators and instructors. They were briefed on the research objectives and subsequently played an essential role in disseminating information about the study to eligible students. Invitations to participate in the research were extended through a combination of email communication and in-person interactions during class sessions. During these engagements, participants received a comprehensive overview of the research, emphasizing the voluntary nature of participation and the strict confidentiality of their responses. In addition, it is important to note that all respondents in our study were intermediate-level EFL students. As such, we employed the English version of the scales. This approach ensured that language consistency was maintained throughout the questionnaire, eliminating the need for translations and potential language-related biases. Furthermore, to establish the validity and robustness of the measurement model, the scales used were revalidated in this study by testing the measurement model and conducting Confirmatory Factor Analysis (CFA).

Data collection was effectively conducted through a structured online questionnaire. Before administering the survey, participants were presented with informed consent information, and their explicit consent was obtained before proceeding. The participants were afforded ample time to complete the survey at their convenience. To preserve data integrity and mitigate potential response biases, participants were assured of the anonymity and confidentiality of their responses. Additionally, reminders were proactively sent to non-respondents to encourage participation and ensure representation across all selected groups.

The data collection phase spanned 4 weeks to accommodate participants’ schedules and ensure a robust response rate. This timeframe was chosen deliberately to facilitate comprehensive data collection while minimizing any undue pressure on participants. During this period, we achieved a response rate of 81%, indicating a high level of engagement and cooperation from the participants. This strong response rate enhances the reliability and validity of our findings, ensuring that the collected data is representative of the study population and providing a solid foundation for our research analysis.

Ethical approval for this research was diligently secured from the relevant ethics committee or institution, aligning with established ethical guidelines governing research involving human participants. Throughout the study’s duration, strict adherence to ethical principles was maintained, encompassing informed consent, voluntary participation, data confidentiality, and participants’ unequivocal right to withdraw from the study at any point without consequences. The data collection process was facilitated through a secure and user-friendly online platform, prioritizing participant convenience and accessibility. The online questionnaire was hosted on a trusted and reputable data collection platform, further fortifying data privacy and security. The research team remained readily accessible to address any queries or concerns raised by participants during the data collection phase. Comprehensive records of participant responses were securely stored and protected, fully aligning with data protection regulations.

### 3.4. Data analysis

Before conducting statistical analysis, the data underwent a thorough examination to identify any outliers and address missing values. Additionally, normality tests were applied for data processing. Subsequently, CFA was utilized to assess the reliability and validity of the questionnaires. Descriptive analysis and Pearson bivariate correlation analysis were carried out using SPSS 26.0. Following this, structural equation modeling (SEM) was employed to evaluate the hypothesized model. In both CFA and SEM, AMOS 26.0 served as the analytical tool of choice. The maximum likelihood (ML) estimation method was selected due to its robustness in providing unbiased estimates, even when dealing with non-normally distributed variables within a sizable sample (Hau and Marsh, 2004).

Model fit was evaluated based on specific criteria: Chi-square divided by degrees of freedom (χ^2^/df) < 3 (Hu and Bentler, 1999), Comparative Fit Index (CFI) and Tucker–Lewis Index (TLI) values ≥ 0.90, and Root Mean Square Error of Approximation (RMSEA) and Standardized Root Mean Square Residual (SRMR) values ≤ 0.08 (Kline, 2011). Finally, mediation effects were assessed through a bootstrap analysis consisting of 5,000 samples, and bias-corrected 95% confidence intervals were generated to ascertain the significance of these effects.

## 4. Results

### 4.1. Measurement model

To establish the validity and robustness of the measurement model, we conducted a Confirmatory Factor Analysis (CFA) utilizing AMOS 26.0. This analytical approach allowed for an assessment of the proposed connections between latent constructs and their observable indicators, as well as an evaluation of the model’s conformity with the data. Initially, the measurement model incorporated multiple observed indicators derived from their respective scales for each of the four latent constructs. However, the initial model’s fit indices did not meet the criteria for a satisfactory fit. Specifically, the fit indices were as follows: χ^2^(243) = 480.213, CFI = 0.921, TLI = 0.917, RMSEA = 0.078, SRMR = 0.063.

To enhance construct validity and overall model fit, we embarked on an iterative refinement process. This involved a meticulous examination of modification indices and a commitment to established guidelines to identify potential sources of model misfit. Two items from the online learning self-efficacy scale displayed low factor loadings, signaling inadequate representation of the underlying construct. Consequently, these items were excluded from the model to bolster construct validity and conceptual coherence. Likewise, one item from the grit scale as well as two items from the online learning satisfaction scale, exhibited weak factor loadings. In the pursuit of improving clarity and unidimensionality within their respective constructs, these items were also removed from the model.

Following these modifications, the revised measurement model underwent another round of CFA analysis. The results of this analysis indicated a substantial improvement in fit indices, affirming enhanced model fit and construct validity. Specifically, the fit indices for the revised model were as follows: χ^2^(276) = 550.283, CFI = 0.962, TLI = 0.965, RMSEA = 0.036, SRMR = 0.042.

A comparative assessment of fit indices between the initial and revised models unequivocally demonstrates that the revised model exhibited significantly superior goodness-of-fit statistics. Given the substantial enhancement in fit indices and the improved construct validity resulting from the modifications, we deemed the revised model suitable for subsequent data analyses, including SEM to scrutinize the hypothesized relationships between the constructs.

### 4.2. Convergent and divergent validity

After that, we assessed both convergent and divergent validity by examining the Average Variance Extracted (AVE) and Composite Reliability (CR) values for each latent construct, as presented in Table 1.

**TABLE 1:** Convergent and divergent validity.

Variables	AVE	CR	1	2	3	4
1. OLSE	0.61	0.88	**0.78**			
2. Grit	0.57	0.92	0.33**	**0.75**		
3. Ideal L2 self	0.69	0.86	0.40***	0.41***	**0.83**	
4. Online satisfaction	0.74	0.83	0.44***	0.27*	0.51***	**0.86**

AVE, average variance extracted; CR, composite reliability. Bold font numbers are square roots of the AVE; off diagonals are correlation coefficients. *p < 0.05; **p < 0.01; ***p < 0.001.

The AVE values, which indicate the proportion of variance captured by the construct’s indicators, exceeded the recommended threshold of 0.5 (Fornell and Larcker, 1981) for all constructs, confirming satisfactory convergent validity. Specifically, the AVE values for online learning self-efficacy (OLSE), grit, ideal L2 self, and online learning satisfaction were 0.61, 0.57, 0.69, and 0.74, respectively. Composite Reliability (CR) values, reflecting the internal consistency of the constructs, surpassed the acceptable threshold of 0.7 (Bagozzi and Yi, 1988) for all constructs, indicating a high degree of reliability. The CR values for OLSE, grit, ideal L2 self, and online learning satisfaction were 0.88, 0.92, 0.86, and 0.83, respectively.

To evaluate discriminant validity, we examined the correlation matrix (Table 1). The square roots of the AVE values, denoted in bold font along the diagonal, exceeded the correlation coefficients in their respective rows and columns. This demonstration of satisfactory discriminant validity (Fornell and Larcker, 1981) reinforces the distinctiveness of the latent constructs.

These results collectively indicate that the constructs in the study exhibit satisfactory convergent and divergent validity, providing a robust foundation for subsequent analyses examining the structural relationships between the latent variables.

### 4.3. Descriptive statistics and reliability indices

Descriptive statistics and reliability indices for each variable in the study are presented in Table 2. Participants reported their perceived online learning self-efficacy (OLSE) with a mean score of 3.38 (*SD* = 0.80), indicating their confidence in online learning. The distribution of responses showed slight negative skewness (−0.11) and kurtosis (0.13), indicating a relatively balanced spread of scores for OLSE.

**TABLE 2:** Descriptive statistics and reliability indices.

Variables	Mean	SD	Skewness	Kurtosis	Cronbach’s alpha
1. OLSE	3.38	0.80	−0.11	0.13	0.83
2. Grit	3.55	0.93	−0.09	0.12	0.84
3. Ideal L2 self	3.09	0.69	−0.06	0.09	0.89
4. Online satisfaction	3.97	0.81	−0.09	0.08	0.91

Moving on to the grit variable, participants demonstrated a moderate level with an average score of 3.55 (*SD* = 0.93). The skewness (−0.09) and kurtosis (0.12) values indicated a generally normal distribution of responses, adding robustness to this variable. The ideal L2 self-scale revealed participants’ vision of their ideal language proficiency, with an average score of 3.09 (*SD* = 0.69). The skewness (−0.06) and kurtosis (0.09) values suggested a relatively symmetrical distribution of scores, further affirming the credibility of this measure. Additionally, the online learning satisfaction variable indicated a relatively high level of satisfaction among participants, with an average score of 3.97 (*SD* = 0.81). The skewness (−0.09) and kurtosis (0.08) values approximated normal distribution of responses.

Moreover, the reliability indices for each measurement scale were computed and affirmed high levels of internal consistency. The OLSE scale demonstrated good reliability with a Cronbach’s alpha coefficient of 0.83, confirming its effectiveness in measuring online learning self-efficacy. Similarly, the Grit scale exhibited good reliability, with a Cronbach’s alpha coefficient of 0.84, indicating its proficiency in capturing participants’ perseverance and passion for long-term goals. The ideal L2 self-scale displayed excellent internal consistency, with a Cronbach’s alpha coefficient of 0.89, reinforcing its validity as a measurement tool for participants’ ideal language proficiency. Lastly, the online learning satisfaction scale demonstrated strong internal consistency, with a Cronbach’s alpha coefficient of 0.91, affirming its ability to measure participants’ satisfaction with online learning experiences.

Table 3 presents the correlations among the latent constructs in the study, shedding light on the relationships between online learning self-efficacy (OLSE), grit, ideal L2 self, and online learning satisfaction. These correlation coefficients reveal the interrelatedness of the studied constructs, highlighting the potential influence of OLSE, grit, and ideal L2 self on online learning satisfaction.

**TABLE 3:** Correlations among the constructs.

Variables	1	2	3	4
1. OLSE	1.00			
2. Grit	0.33**	1.00		
3. Ideal L2 self	0.40***	0.41***	1.00	
4. Online satisfaction	0.44***	0.27*	0.51***	1.00

*p < 0.05, **p < 0.01, ***p < 0.001.

Online learning self-efficacy exhibited significant positive correlations with the other constructs. The correlation with grit was moderate (r[494] = 0.33*, p* < 0.01), indicating a positive relationship. Similarly, OLSE correlated positively with ideal L2 self (r[494] = 0.40, *p* < 0.001) and online learning satisfaction (r[494] = 0.44, *p* < 0.001), demonstrating moderate to strong positive relationships. Grit demonstrated significant positive correlations with OLSE (r[494] = 0.33, *p* < 0.01), ideal L2 self (r[494] = 0.41, *p* < 0.001), and online learning satisfaction (r[494] = 0.27, *p* < 0.05). These correlations indicate moderate positive relationships, suggesting that students with higher levels of tenacity also tend to have higher self-efficacy and report greater online learning satisfaction.

Ideal L2 self-exhibited significant positive correlations with OLSE (r[494] = 0.40, *p* < 0.001), grit (r[494] = 0.41, *p* < 0.001), and online learning satisfaction (r[494] = 0.51, *p* < 0.001). These correlations signify moderate to strong positive relationships, implying that students who strongly visualize their ideal language proficiency also tend to display higher levels of grit, self-efficacy, and satisfaction with online learning. Online learning satisfaction demonstrated significant positive correlations with OLSE (r[494] = 0.44, *p* < 0.001), grit (r[494] = 0.27, *p* < 0.05), and ideal L2 self (r[494] = 0.51, *p* < 0.001). These correlations indicate moderate to strong positive relationships, emphasizing that students who report higher satisfaction with online learning also tend to have greater self-efficacy, grit, and a clear vision of their ideal language proficiency.

These findings provide a foundation for the subsequent structural equation modeling (SEM) analysis to explore the hypothesized relationships among OLSE, grit, ideal L2 self, and online learning satisfaction in greater detail.

### 4.4. SEM analysis

Then SEM was employed to scrutinize the hypothesized relationships between the constructs. The model fit indices revealed a commendable alignment between the proposed model and the observed data, as evidenced by the following results: χ^2^(347) = 460.79, p = 0.001, CFI = 0.977, TLI = 0.972, RMSEA = 0.032, 95% CI = 0.029–0.035.

**FIGURE 2:**
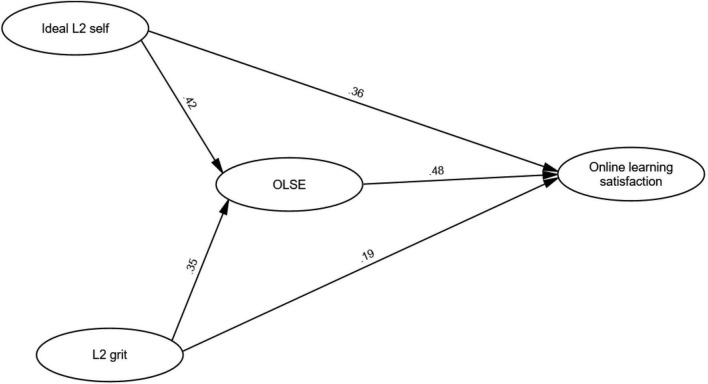
The mediation model.

Figure 2 displays the path diagram, illustrating the hypothesized relationships between the latent constructs. Notably, all path coefficients were found to be statistically significant, lending robust support to the expected associations between the variables. Specifically, ideal L2 self-exhibited a positive relationship with online learning satisfaction (β = 0.36), affirming that students who held a stronger ideal L2 self-tended to report higher levels of satisfaction with online learning experiences. Moreover, ideal L2 self was positively related to OLSE (β = 0.42), indicating that a more vivid ideal L2 self-concept was associated with a greater sense of self-efficacy in the context of online learning. Similarly, grit demonstrated a significant positive relationship with Online Learning Satisfaction (β = 0.19), signifying that students with higher levels of grit tended to report elevated levels of satisfaction with their online learning experiences.

Furthermore, grit exhibited a positive relationship with OLSE (β = 0.35), highlighting that individuals with greater grit were more likely to possess enhanced self-efficacy in online learning tasks. In addition, OLSE was significantly related to online learning satisfaction (β = 0.48), emphasizing the importance of self-efficacy beliefs in influencing students’ satisfaction with their online learning experiences.

These SEM results provide empirical evidence for the hypothesized associations between the latent constructs and offer valuable insights into the factors contributing to online learning satisfaction among the study’s participants.

### 4.5. Mediation analysis

To ascertain the significance of the indirect effects, bootstrapping analyses were conducted employing 5,000 resamples, following Hayes (2013) guidelines. The results of the bootstrapping analyses are summarized in Table 4, presenting the direct, indirect, and total effects in the mediation analysis.

**TABLE 4:** Direct, indirect, and total effects in mediation analysis.

Path	Coefficient (β)	Bootstrapped 95% CI	*p*-value	Effect type
Ideal L2 self → Satisfaction	0.36	[0.30, 0.42]	<0.001	Direct
Grit → Satisfaction	0.19	[0.14, 0.24]	<0.001	Direct
OLSE → Satisfaction	0.48	[0.43, 0.53]	<0.001	Direct
Ideal L2 self → OLSE → Satisfaction	0.20	[0.15, 0.25]	<0.001	Indirect
Grit → OLSE → Satisfaction	0.16	[0.11, 0.21]	<0.001	Indirect
Ideal L2 self → Satisfaction (Total)	0.56	[0.50, 0.62]	<0.001	Total
Grit → Satisfaction (total)	0.35	[0.30, 0.40]	<0.001	Total

Satisfaction: online learning satisfaction, OLSE: online learning self-efficacy, Bootstrap is based on 5,000 resamples (Hayes, 2013).

The results of the mediation analysis, as presented in Table 4, provide a comprehensive understanding of the various effects within the model, shedding light on the intricate relationships between the constructs.

Firstly, the direct effects in the paths from ideal L2 self, grit, and OLSE to online learning satisfaction are statistically significant, with coefficients of 0.36, 0.19, and 0.48, respectively (all *p*-values < 0.001). These findings underscore the individual impact of each construct on online learning satisfaction. In other words, students with a stronger ideal L2 self, higher levels of grit, and greater online learning self-efficacy tend to report higher levels of satisfaction with their online learning experiences.

Furthermore, the mediation analysis uncovers significant indirect effects. Specifically, the paths “Ideal L2 Self → OLSE → Satisfaction” and “Grit → OLSE → Satisfaction” demonstrate indirect effects with coefficients of 0.20 and 0.16, respectively (both *p*-values < 0.001). These results highlight the crucial mediating role played by OLSE in the relationship between ideal L2 self and online learning satisfaction, as well as between grit and online learning satisfaction. In essence, OLSE acts as a bridge that partially explains how ideal L2 self and grit influence students’ satisfaction with online learning.

Additionally, the total effects, which encompass both direct and indirect influences, are summarized in the paths “Ideal L2 self → Satisfaction (Total)” and “Grit → Satisfaction (Total),” with coefficients of 0.56 and 0.35, respectively (both *p*-values < 0.001). These total effects provide a holistic view of the combined impact of Ideal L2 Self and Grit on Online Learning Satisfaction, taking into account both the direct effects and the mediating role of OLSE.

## 5. Discussion

The present study aimed to investigate the intricate relationship between ideal L2 self and L2 grit as predictors of online learning satisfaction, with a focus on the mediating role of online learning self-efficacy in Chinese EFL learners. Our findings revealed several noteworthy insights that contribute to our understanding of these relationships.

Firstly, it was found that there is a positive relationship between the ideal L2 self and online learning self-efficacy. This observation aligns with prior research, including the work of Ueki and Takeuchi (2013) and Teng and Yang (2023), indicating that learners who harbor a strong sense of their ideal language proficiency are more likely to possess higher self-efficacy beliefs regarding their online learning abilities. This consistency with Ushioda (2011) conceptualization of the qualitative connection between self-efficacy and the ideal L2 self underscores the robust nature of this relationship within the domain of language learning motivation.

To further support this positive association, we can draw on Bandura (2001) research on self-efficacy, which emphasizes how self-efficacy enhances learners’ motivated learning behaviors. In the context of L2 learning, students who vividly envision their ideal language selves tend to believe more strongly in their capacity to navigate the challenges of online learning (Fathi and Hejazi, 2023). This belief, in turn, motivates them to engage in proactive learning behaviors, such as effective goal setting, resource utilization, and self-regulation (Zimmerman and Kulikowich, 2016). Essentially, the ideal L2 self serves as a guiding beacon, propelling learners toward the achievement of their linguistic aspirations, with online self-efficacy playing a vital catalytic role in this process (Fathi et al., 2023). Moreover, we can interpret the relationship between the ideal L2 self and online learning self-efficacy through the lens of self-regulation theory (Zimmerman, 2002). Learners who possess a distinct and compelling vision of their ideal language proficiency are better equipped to assess the discrepancy between their current language abilities and their ideal selves (Dörnyei, 2009). This heightened awareness of the gap between their present and desired linguistic competencies empowers students to set clear learning objectives, formulate effective strategies, and exert the necessary effort to bridge this divide. In contrast, learners with a less vivid ideal L2 self-image may struggle to accurately gauge the extent of this disparity, potentially lacking the motivation and direction needed to effectively narrow the gap (Adolphs et al., 2018).

Secondly, our study unveiled a positive association between L2 grit and online learning self-efficacy. This finding indicates that learners who display greater levels of perseverance, passion, and dedication in their language learning endeavors tend to develop higher levels of confidence in their ability to effectively navigate online learning environments (Paradowski and Jeliñska, 2023; Zhao et al., 2023). This observed link between self-efficacy beliefs and tenacity in learning, followed by enthusiasm for learning, aligns with the theoretical foundations of grit as proposed by Duckworth et al. (2007). Grit, characterized by sustained effort and persistence toward long-term goals, inherently involves the development of self-efficacy in one’s abilities (Teimouri et al., 2022). As individuals consistently engage in challenging tasks and persevere through difficulties, they acquire firsthand evidence of their competence, which, in turn, fosters higher levels of self-efficacy (Bandura, 1989). This cyclical relationship between grit and self-efficacy gains particular significance in the context of online learning, where learners encounter various challenges and uncertainties (Alhadabi and Karpinski, 2020; Neroni et al., 2022).

It is worth noting that higher self-efficacy beliefs have consistently been associated with enhanced performance and greater persistence in the face of obstacles (Bandura, 2001). Learners who possess strong self-efficacy beliefs are more likely to actively engage in online instruction and persist in their learning efforts, even when encountering difficulties. This heightened engagement contributes to a more fruitful and satisfying online learning experience, as students are motivated to invest more effort and time in their studies (Zimmerman and Kulikowich, 2016). Furthermore, our findings align with previous research conducted by Derakhshan and Fathi (2023), who discovered that hardworking EFL students with self-efficacy attitudes in online learning environments tend to be more actively engaged in the learning process. Given that online language learning environments pose unique challenges, such as shifts in roles and responsibilities, the integration of technology, and the formation of new interpersonal relationships (Montano, 2021), these challenges may appear particularly daunting for EFL students who lack sufficient online learning self-efficacy. However, learners with higher levels of grit are better equipped to confront and overcome these unforeseen difficulties. Their inherent resilience and perseverance enable them to adapt to changing circumstances and persist in their online language learning process (Duckworth and Gross, 2014).

Thirdly, our study uncovered a direct and positive relationship between the ideal L2 self and online learning satisfaction among EFL students. This outcome underscores the pivotal role of self-concept and motivation in the context of language learning and reinforces the existing literature’s emphasis on the significance of learners’ ideal selves in shaping their motivation and engagement (Dörnyei, 2005; Ushioda, 2009; Dörnyei and Ushioda, 2011). The direct link between the ideal L2 self and online learning satisfaction implies that the fulfillment of this motivational construct is intrinsically tied to the quality of the online learning experience (Ji et al., 2022). When EFL students perceive that their online language courses contribute to the realization of their ideal L2 selves, they are more inclined to find satisfaction in the learning process. This finding aligns with earlier studies indicating that learner motivation and positive self-concepts are closely linked to higher satisfaction levels in educational settings (Elliot and Dweck, 1988; Deci et al., 1991; Zhan and Mei, 2013; Bringula et al., 2021). Furthermore, the online learning environment can offer unique opportunities for learners to envision and work toward their ideal L2 selves. Online courses often provide flexible schedules, diverse learning resources, and opportunities for self-directed study—all of which can empower EFL students to tailor their learning experiences in alignment with their ideal language proficiency goals (Wong and Bakar, 2009; Adolphs et al., 2018). This alignment between the online learning environment and learners’ motivational constructs may further enhance satisfaction. Importantly, the relationship between the ideal L2 self and online learning satisfaction is also consistent with the broader literature on self-determination theory (Deci and Ryan, 2000). According to this theory, individuals who feel autonomous in their pursuits, experience a sense of competence, and have a clear sense of relatedness to their goals are more likely to be intrinsically motivated and, consequently, satisfied. The ideal L2 self-embodies these elements by fostering learners’ autonomy in pursuing their language learning goals and enhancing their perceived competence in language acquisition (Ueki and Takeuchi, 2013).

Fourthly, our study revealed a positive and direct relationship between L2 grit and online learning satisfaction among EFL students. This finding substantiates the critical role that grit plays in shaping students’ experiences and outcomes in online language learning environments (Zarrinabadi et al., 2022). It is in line with previous research that has recognized the significance of grit as a determinant of academic achievement, motivation, and positive emotions (Vainio and Daukantaitë, 2016; Datu et al., 2017; Jiang et al., 2020; Derakhshan and Fathi, 2023). Furthermore, it underscores the value of considering grit in the context of language learning, especially in the online domain.

The connection between L2 grit and online learning satisfaction aligns with the broader literature on grit’s impact on educational attainment (Elahi Shirvan et al., 2021; Fathi and Hejazi, 2023). Grit’s essence, characterized by perseverance and passion for long-term goals despite adversity, is particularly relevant in the context of online education. EFL students engaging in online learning often face challenges such as self-regulation, self-motivation, and the absence of immediate feedback and social interaction (Artino, 2009; Cho and Cho, 2017; Zhao et al., 2023). Grit equips students with the determination and resilience necessary to navigate these challenges successfully. A noteworthy aspect of this relationship is the concept of passion for long-term goals. Gritty individuals do not merely endure challenges; they do so with a sense of purpose and passion for their objectives (Duckworth et al., 2007). In the context of EFL learning, this passion may translate into a deep commitment to language acquisition, fostering sustained engagement with online courses. As students persist in their efforts to master a new language, they may derive a sense of fulfillment and accomplishment, contributing to their overall satisfaction with the online learning experience (Paradowski and Jeliñska, 2023).

Furthermore, the positive association between L2 grit and online learning satisfaction echoes the idea that grit is not solely related to academic performance but also extends to emotional wellbeing and overall satisfaction (Von Culin et al., 2014; Hill et al., 2016; Datu, 2021). Gritty individuals tend to approach challenges with a growth-oriented perspective, attributing positive outcomes to their efforts and maintaining a sense of agency (Duckworth et al., 2007). In the context of online language learning, this outlook can foster a positive and fulfilling learning experience, thus enhancing satisfaction (Neroni et al., 2022).

Fifthly, our study revealed that online learning self-efficacy (OLSE) played a mediating role in the relationship between the ideal L2 self and online learning satisfaction among EFL students. This result aligns with existing literature emphasizing the role of self-efficacy as a mediator of motivation and satisfaction (Bandura, 1977; Zimmerman and Kulikowich, 2016). In the context of language learning, OLSE encompasses learners’ confidence in their ability to navigate digital tools, engage with online materials, and manage their own learning effectively in the online setting (Derakhshan and Fathi, 2023). Given the unique challenges of online language learning, including the need for self-regulation and technology proficiency, OLSE plays a pivotal role in determining learners’ success and satisfaction in this environment (Shen et al., 2013; Cho and Cho, 2017; Derakhshesh et al., 2022).

The mediating role of OLSE suggests that learners with a strong ideal L2 self are more likely to invest effort in online language learning and, in turn, develop higher levels of OLSE. This is in line with Bandura (1977) theory of self-efficacy, which posits that beliefs in one’s abilities are influenced by personal goals and previous experiences. As EFL students strive to achieve their ideal L2 selves, they are motivated to develop the necessary skills and strategies to succeed in the online language learning environment, thus enhancing OLSE. OLSE, as a mediator, bridges the gap between motivation (ideal L2 self) and satisfaction. Learners with high OLSE are better equipped to engage effectively with online courses, navigate challenges, and adapt to the digital learning landscape. As a result, they are more likely to experience satisfaction with the online learning experience (Womble, 2007). This finding is consistent with studies highlighting the importance of self-efficacy in predicting online learning satisfaction (Shen et al., 2013; Arpaci, 2017; Zhu et al., 2020).

Finally, OLSE was found to act as a mediator in the relationship between L2 grit and online learning satisfaction among EFL students. This finding aligns with existing literature emphasizing the significance of self-efficacy in mediating the impact of personal traits and motivation on learning outcomes (Bandura, 1977; Zimmerman and Kulikowich, 2016; Cerezo et al., 2019; Wu et al., 2020). For EFL students engaged in digital language learning, OLSE encompasses their confidence in using digital tools, effectively engaging with online language resources, and managing their own learning autonomously (Han et al., 2021). Given the unique challenges of online language learning, including the need for self-regulation and technological proficiency, OLSE plays a pivotal role in determining learners’ success and satisfaction in this environment (Shen et al., 2013; Cho and Cho, 2017; Derakhshesh et al., 2022).

The mediating role of OLSE in the relationship between L2 grit and online learning satisfaction suggests a nuanced pathway to satisfaction in digital language education. Learners with high levels of L2 grit demonstrate a strong determination to persist in their language learning endeavors, even when facing adversity or plateaus in progress (Teimouri et al., 2022). This determination, while essential, may not directly translate into satisfaction unless learners possess the necessary self-efficacy beliefs to effectively navigate the online learning environment (Derakhshan and Fathi, 2023). OLSE, as a mediator, bridges the gap between the perseverance and passion represented by L2 grit and the actual experience of satisfaction in the online language learning context. Learners with elevated levels of L2 grit are more likely to invest effort and maintain interest in language learning over time, ultimately leading to greater OLSE. This enhanced self-efficacy empowers learners to tackle online language courses with confidence, effectively manage challenges, and engage more actively, ultimately resulting in higher levels of satisfaction with the online learning experience (Derakhshesh et al., 2022). This finding echoes the insights provided by studies highlighting the critical role of self-efficacy in predicting online learning satisfaction (Puzziferro, 2008; Shen et al., 2013; Alqurashi, 2016; Zhu et al., 2020). It also underscores the interconnectedness of motivational constructs and learner attributes in the digital language learning realm. Educators and instructional designers should consider not only learners’ motivational profiles but also their perceived self-efficacy when designing online language courses. Strategies aimed at boosting OLSE, such as providing comprehensive digital literacy support and scaffolding learners’ online learning skills, may be essential for optimizing satisfaction and success in digital language education.

## 6. Conclusion

In conclusion, this study delved into the complex web of factors influencing online learning satisfaction among EFL students. The findings revealed important insights into the roles of ideal L2 self, L2 grit, and online learning self-efficacy in shaping students’ satisfaction with the online language learning experience. The direct positive relationship between ideal L2 self and online learning satisfaction underscores the significance of learners’ aspirations and visions in influencing their satisfaction levels. When students can envision their future selves proficiently communicating in a second language, it propels them toward greater satisfaction with their online language learning process. Similarly, the direct positive relationship between L2 grit and online learning satisfaction highlights the importance of perseverance and passion in achieving satisfaction in the face of challenges. Students who exhibit grit are more likely to persist in their language learning efforts and, as a result, experience higher levels of satisfaction. The mediating role of online learning self-efficacy in both relationships signifies the pivotal role of learners’ beliefs in their ability to succeed in online language courses. Online learning self-efficacy serves as a bridge connecting students’ ideal L2 self and grit to their ultimate satisfaction. When students possess the confidence and self-belief to tackle online learning challenges, it enhances their grit and satisfaction levels.

The findings of this study hold significant implications for various stakeholders involved in the realm of online language education. Educators and course designers are urged to consider the motivational elements of the ideal L2 self and grit when crafting online language courses. By integrating components that enable students to envision their future language proficiency and by fostering grit through the incorporation of challenging yet attainable objectives, educators can augment levels of satisfaction and motivation among learners. Pedagogical approaches within online language learning platforms stand to benefit from strategies aimed at enhancing online learning self-efficacy. Practices such as providing lucid instructions, facilitating peer support networks, and offering timely and constructive feedback are instrumental in bolstering learners’ confidence. This, in turn, contributes to an elevation in overall satisfaction levels with the online learning experience.

Teacher training programs should prioritize equipping instructors with the knowledge and techniques necessary to cultivate grit and self-efficacy in the context of online language learning. Educators, armed with the tools to nurture students’ growth mindset and perseverance, assume a crucial role in fortifying satisfaction levels. Institutions offering online language education should consider implementing robust student support services that specifically target the enhancement of self-efficacy beliefs and resilience. Programs such as academic advising, counseling, and mentoring play pivotal roles in aiding students as they navigate the challenges inherent in online language learning. These services ultimately serve to sustain students’ motivation and satisfaction throughout their learning journey. For future research endeavors, it is imperative to delve deeper into the intricate dynamics of ideal L2 self, grit, and self-efficacy in the context of online language learning. Exploring additional moderators and mediators within this multifaceted relationship will yield a more nuanced understanding of how to optimize satisfaction levels in online language education. By delving into these aspects, researchers can contribute further insights that will refine instructional practices and advance the overall effectiveness of online language learning platforms.

This study, while yielding valuable insights into the factors that influence online learning satisfaction among EFL students, is not without its limitations. First and foremost, the study employed a cross-sectional design, a method that inherently limits our capacity to definitively establish causal relationships among the variables under investigation. To enhance the depth of our comprehension regarding the complex interplay between ideal L2 self, grit, online learning self-efficacy, and satisfaction, future research should consider embracing longitudinal or experimental designs, which offer a more robust platform for analysis and inference.

Secondly, the study’s exclusive focus on intermediate-level EFL students in mainland China implies a limitation in terms of generalizability. The findings may not necessarily apply to learners at different proficiency levels or those hailing from diverse cultural backgrounds. To ensure broader insights, it is advisable to replicate this research with a more diverse and representative sample. Thirdly, data collection relied heavily on self-report measures, which are susceptible to response biases, including social desirability and recall bias. The incorporation of objective measures or observational data in future studies could enhance the overall validity of the findings. Moreover, while this study explored the mediating role of online learning self-efficacy, it is possible that other unexamined mediators influence the relationships between ideal L2 self, grit, and satisfaction. To attain a more comprehensive understanding, future research should consider additional mediating variables. Furthermore, this study did not delve deeply into the contextual factors that can significantly vary within online language learning environments, such as the platform used, instructional methods, and course content. These contextual factors were not thoroughly examined, and future research should aim to consider the influence of specific course characteristics.

Additionally, this study primarily concentrated on cognitive factors, including ideal L2 self, grit, and self-efficacy, as influencing satisfaction. The roles of social support, emotional wellbeing, and affective factors in online language learning satisfaction remain a pertinent avenue for future investigation. While the findings have clear implications for online language learning contexts, caution should be exercised when attempting to generalize the results to other fields or educational settings, as the dynamics of satisfaction may differ significantly in diverse educational domains. In addition, it is important to note that the use of the English version of the scales, rather than a translated version, may limit the generalizability of the findings. For future research, it may be beneficial to explore the applicability of these constructs using the Chinese versions of the scales, allowing for a more precise understanding of their relationships within the local context. Lastly, the possibility of common method bias should be acknowledged, given that all data were collected through self-report surveys. Future studies may benefit from the use of more diverse data collection methods to mitigate this potential limitation.

## Data availability statement

The raw data supporting the conclusions of this article will be made available by the authors, without undue reservation. Requests to access these datasets should be directed to ZS celiasun1030@163.com.

## Ethics statement

The studies involving humans were approved by the Department of Foreign Language, Lanzhou University of Finance and Economics, Lanzhou, 730000, China. The studies were conducted in accordance with the local legislation and institutional requirements. The participants provided their written informed consent to participate in this study.

## Author contributions

ZS: Conceptualization, Data curation, Formal analysis, Funding acquisition, Investigation, Methodology, Project administration, Resources, Software, Supervision, Validation, Visualization, Writing – original draft, Writing – review and editing. BM: Conceptualization, Data curation, Formal analysis, Funding acquisition, Investigation, Methodology, Project administration, Resources, Software, Supervision, Validation, Visualization, Writing – original draft, Writing – review and editing.
